# Is there any geometrical information in the nervous system?

**DOI:** 10.3389/fncom.2013.00121

**Published:** 2013-08-30

**Authors:** Sajad Jafari, Seyed M. R. Hashemi Golpayegani, Shahriar Gharibzadeh

**Affiliations:** Biomedical Engineering Faculty, Amirkabir University of TechnologyTehran, Iran

**Keywords:** trains of impulses, chaotic systems, sensitivity to initial conditions, geometry, phase space

There has been an increasing interest in analyzing neurophysiology from complex and chaotic systems viewpoint in recent years. For example, although the famous Hodgkin and Huxley model (Hodgkin and Huxley, 1952) has been the basis of almost all of the proposed models for neural firing, the Rose-Hindmarsh model (Hindmarsh and Rose, 1984) is known to be a more refined model because as it has the ability of showing different firing patterns, especially chaotic bursts of action potential, which causes a proper matching between this model behavior and many real experimental data.

It is believed that information is transferred in the brain by trains of impulses, or action potentials, often organized in sequences of bursts; therefore, it is useful to determine the temporal patterns of such trains (Korn and Faure, 2003). Since chaotic systems are sensitive to initial conditions (Hilborn, 2000), lots of signals with minimum similarity in time domain could have a same source; such behavior might be better understood by analyzing those signals in the phase space and from geometrical viewpoint (Jafari et al., 2013d), as although chaotic signals have pseudorandom behavior in time, they are ordered in phase space (i.e., if one plots the signals as a trajectory in a coordinate of system variables, he will encounter an ordered and specific topology which is called strange attractor) (Hilborn, 2000).

In fact in many applications of chaotic signals and systems, using temporal properties without being careful about this sensitivity to initial conditions, could lead to important misinterpretations (Jafari et al., 2012, 2013a,c,d). Hence, it seems that more than temporal patterns, it is of paramount importance to investigate topological patterns in such impulse trains. In order to accomplish such tasks several we have recently proposed some interesting tools for geometrical analysis (Jafari et al., in press; Shekofteh et al., in press).

In order to show the benefit of using geometry and topology in the phase space (state space), a simple example is provided in the sequence. Consider the famous Logistic map which is a very simple and well investigated chaotic map:

(1) $$x_{k+1}=Ax_{k}\left(1-x_{k}\right)$$

Suppose that we have two different maps with different values of parameter A:

(2) $$\begin{array}{l} x_{k+1}=\text{3}.\text{8}x_{k}\left(1-x_{k}\right)\\ \;x_{k\text{+1}}=3.9x_{k}\left(1-x_{k}\right) \end{array}$$

**Figure 1:**
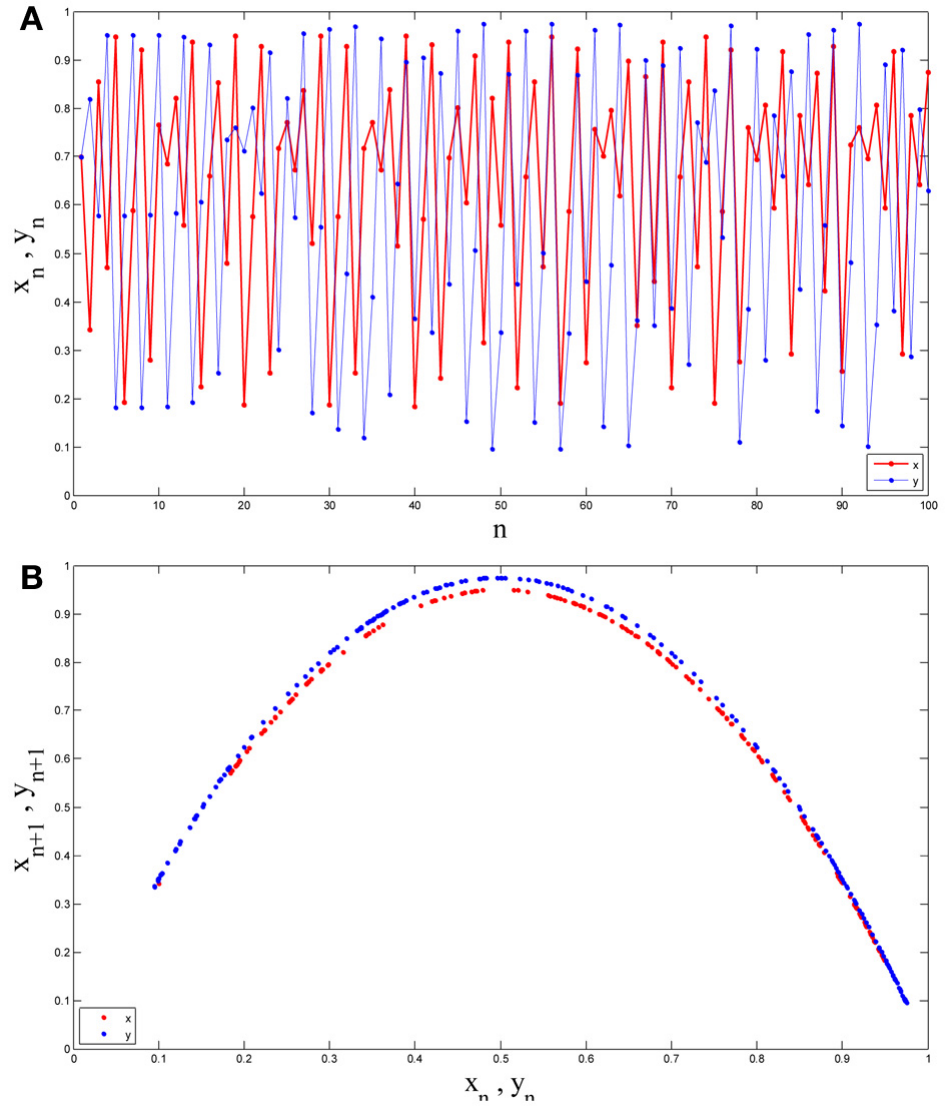
**(A)** Two time series obtained from two different Logistic maps. **(B)** Those two time series embedded in the state space. As can be seen while recognizing the difference between them is not such easy in the time domain (both are random-like), they have two ordered and easily distinguishable pattern in the state space.

If we obtain one time series from each of them, as can be seen in Figure 1A, they are both random-like and recognizing the difference between them seems difficult in the time domain. However, they have two ordered and easily distinguishable patterns in the state space (Figure 1B).

Since looking at neurophysiology from dynamical and geometrical points of view has already been successfully investigated in some previous works (Sauer, 1994; Christini and Collins, 1995; Gottschalk et al., 1995; Milton and Black, 1995; Sarbadhikari and Chakrabarty, 2001; Korn and Faure, 2003; Hadaeghi et al., 2013; Jafari et al., 2013a), we believe that future investigations, especially using real clinical data, will be able evaluate our hypothesis and prove the benefit of such geometrical analysis of non-linear data. Ultimately, a better understanding of neuronal information transportation from the nonlinear dynamics standpoint is expected to provide a better understanding of the basic pathophysiology of neurological disorders, possibly fostering new future therapeutic approaches.
